# Detemplated and Pillared 2-Dimensional Zeolite ZSM-55 with Ferrierite Layer Topology as a Carrier for Drugs

**DOI:** 10.3390/molecules25153501

**Published:** 2020-07-31

**Authors:** Weronika Strzempek, Aleksandra Korzeniowska, Andrzej Kowalczyk, Wieslaw J. Roth, Barbara Gil

**Affiliations:** Faculty of Chemistry, Jagiellonian University, Gronostajowa 2, 30-387 Kraków, Poland; weronika.strzempek@doctoral.uj.edu.pl (W.S.); aleksandra.korzeniowska@uj.edu.pl (A.K.); kowalczy@chemia.uj.edu.pl (A.K.); wieslaw.roth@uj.edu.pl (W.J.R.)

**Keywords:** 2D zeolites, ZSM-55, drug delivery, modified release, piracetam, ciprofloxacin

## Abstract

The present studies were conducted to show the potential of 2D zeolites as effective and non-toxic carriers of drugs. Layered zeolites exhibit adjustable interlayer porosity which can be exploited for controlled drug delivery allowing detailed investigation of the drug release because the structure of the carrier is known exactly. This study was conducted with model drugs ciprofloxacin and piracetam, and ZSM-55 with ca 1 nm thick layers, in detemplated and pillared forms. The release profiles differed from the commercial, crystalline forms of drugs—the release rate increased for ciprofloxacin and decreased for piracetam. To understand the dissolution mechanisms the release data were fitted to Korsmeyer-Peppas equation, showing Fickian (for pillared) and anomalous (for detemplated sample) transport. FT-IR studies showed that strong interaction carrier-drug may be responsible for the modified, slowed down release of piracetam while better solubility and faster release of ciprofloxacin was attributed to formation of the protonated form resulting in weaker interaction with the zeolite than in the pure crystalline form. Two independent tests on L929 mice fibroblasts (ToxiLight and PrestoBlue) showed that ZSM-55, in moderate concentrations may be safely used as a carrier of drug molecules, not having negative effect on the cells viability or proliferation rate.

## 1. Introduction

One of the principal challenges of modern pharmaceutical technology is drug formulation aimed not only at the convenience of use, but also providing the optimal concentration of a drug at the site of action [1,2,3,4,5,6,7]. In an ideal situation, the concentration should rapidly increase right after the application/ingestion and maintain a constant level over the time period required to achieve the desired therapeutic effect; afterwards the drug should be eliminated from the body [8,9]. Inert supports, carriers, are used to deliver therapeutic substances and then release them at the right moment and over a period of time sufficient to achieve the therapeutic goal. There is an ongoing effort in the area of materials to generate new carrier solids with high drug capacity and selectivity depending on the properties of molecules being adsorbed, i.e., guests [1,3,10,11].

The release of therapeutic molecules from porous matrices may occur by different mechanisms. The extreme cases are, at one end, slow degradation of the drug-carrier composite initiated by surface erosion or, at the other end, simple diffusion of drug molecules out of the host matrix depending on its porous structure [12]. The most common mechanism combines both cases [13] and consequently the process of drug release is difficult to characterize. Considering this, we wanted to examine modes of interaction between the carrier and drug molecules, since the nature of their interplay influences subsequent process of drug release. The model carrier materials chosen for this study, namely 2-dimensional (2D) zeolites, which are a relatively new class of layered materials that are especially suited for that purpose. They consist of layers with thicknesses not greater than a few nanometers with internal structures (topologies) of zeolites. The typical forms of 2D zeolites are analogous to other classes of layered materials, namely stacks of layers equally spaced with separations depending on intercalated guest compounds. These structures have been visualized by TEM as illustrated by many examples in the literature [14,15,16]. 2D zeolites possess interlayer spaces large enough to accept bulky guest molecules and well-defined structures and surfaces allowing examination of the drug-carrier interactions [17,18,19]. The reports of 2D zeolites investigations as carriers for therapeutic substances are practically non-existent in the literature, and to the best of our knowledge, there have been no articles dealing directly with this problem so far.

The carrier chosen for the testing of storage and release of drugs, is designated ZSM-55. It is a borosilicate composed of layers with FER (zeolite ferrierite) topology with embedded choline cations serving as a template, organic structure directing agent, during the synthesis. ZSM-55 condenses upon calcination to produce the 3D zeolite framework designated CDO with unidimensional 8-ring pores, thus is formally a layered precursor to zeolite CDO [20]. Its interlayer space and pore structure can be modified by detemplation (interlayer template extraction with an acid), swelling and then pillaring [21,22]. According to the literature one of the most important factors influencing the drug-carrier interactions is the surface topography of the carrier [23]. There were two main reasons for choosing ZSM-55 zeolite as a carrier. Firstly, the ferrierite layers with thickness 0.9 nm are non-porous, thus the interaction with a drug can only take place in the interlayer spaces, which simplifies interpretation of the adsorption and release processes. Secondly, we wanted to compare different types of supports based on the same layer: detemplated and pillared. In the case of detemplated material, the interlayer space may be flexible as the ferrierite layers are linked by hydrogen bonds which have to be broken to accommodate drug molecules. Upon intercalation, the layers are separated with the interlayer distance depending on the size and amount of introduced molecules (drug). For the pillared form, the interlayer distance is fixed and should not change upon drug introduction or release, as long as the zeolite matrix is not damaged, which is the most likely scenario—the pillaring is complete upon calcination at over 500 °C leading to a very robust porous material.

Two drugs of low bioavailability but differing in their solubility in water and body fluids were chosen: ciprofloxacin, representing poorly soluble substance and piracetam, representing highly soluble drug. In both cases, formation of composites based on a zeolite support would allow the drug to gradually pass into solution, increasing its bioavailability.

**Figure 1:**
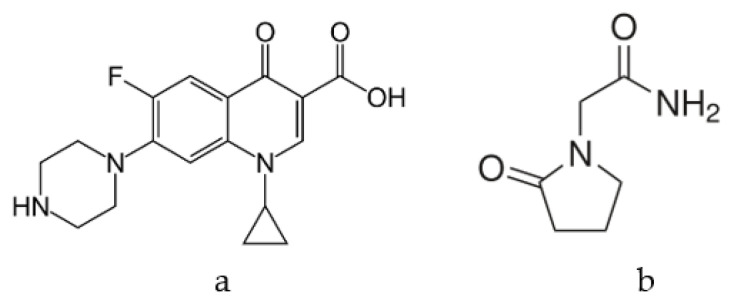
Ciprofloxacin (**a**) and piracetam (**b**) molecules.

Ciprofloxacin (Figure 1a) is a representative of the fluoroquinolones, which are broad-spectrum antibiotics for both Gram-positive and Gram-negative bacteria, hence used to combat many types of infections and inflammation. Antibiotics of this type are used in the treatment of lower respiratory tract diseases and skin inflammation [24]. Ciprofloxacin (molecular mass 331.35 g/mol) is practically insoluble in water (<1 mg/mL) [25,26] but its solubility is improved by formation of salts (citrates, tartrates, malonates, succinates), since ionic compounds are more easily solvated (hydrated) compared to neutral forms [25,26,27]. Alternatively, as proposed here, ciprofloxacin solubility may be increased by formation of alternative interactions between a high-surface carrier and ciprofloxacin molecules.

Ciprofloxacin is available in forms of oral tablets, ophthalmic (eye drops), otic (ear drops) or oral and intravenous suspensions. The oral or intravenous route of administration, although very simple, have serious limitations, the most important is first-pass effect and numerous side effects (vomiting, headache, dizziness, hallucinations, convulsions) which preclude administration to a substantial number of patients. Ciprofloxacin is also a well-established broad-spectrum antibiotic indicated for the treatment of exacerbations of respiratory tract infection, especially in Chronic Obstructive Pulmonary Disease (COPD), cystic fibrosis and bronchiectasis [28]. Unfortunately, the classic ways of administration, due to inter-individual variability in ciprofloxacin pharmacokinetics leads to inadequate drug levels and suboptimal pharmacodynamic exposure, which usually is prevented by increase of the daily dose, escalating the frequency and severity of the side effects. Inhalable application allows for individualization of the dosing to optimize efficacy and to prevent development of resistance [29].

Piracetam (2-(2-oxopyrrolidinyl) acetamide, molecular mass 142.16 g/mol, Figure 1b) is a nootropic drug from the pyrrolidone group [10], with neuroprotective and anticonvulsant properties. Its efficacy is documented in cognitive disorders and dementia [30] and is also used to aid with the learning difficulties. It exhibits linear and time-dependent pharmacokinetic properties with low inter-individual variability over a wide dose range and is absorbed quickly and extensively after oral administration [31,32]. However, approximately 80–100% of the total piracetam dose is excreted in the urine, 90% of which is unchanged (not-metabolized) [33]. Piracetam is a model molecule for drugs with relatively small sizes, a fast release profile and relatively good solubility (72 mg/mL) [34].

The present studies were conducted to show the potential of 2D zeolites as effective and non-toxic carriers of drugs, both of hydrophilic and hydrophobic character.

## 2. Results and Discussion

**Figure 2:**
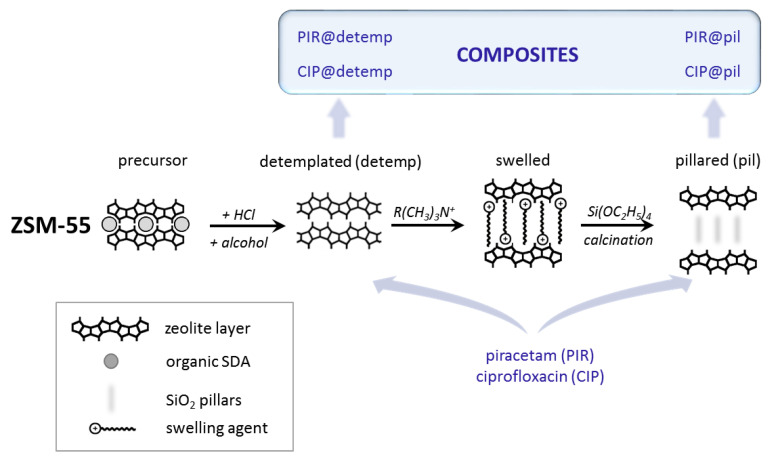
Experiment scheme showing the individual stages of ZSM-55 modifications together with abbreviations used in the manuscript.

Piracetam and ciprofloxacin solutions were contacted with two forms of ZSM-55: acid treated detemplated material with layers connected by weak hydrogen bonds and the expanded pillared form with interlayer pores generated by silica props (pillars) introduced by the standard pillaring with TEOS (tetraethylorthosilicate) and calcination [14]. The scheme of ZSM-55 modification is presented in Figure 2.

**Figure 3:**
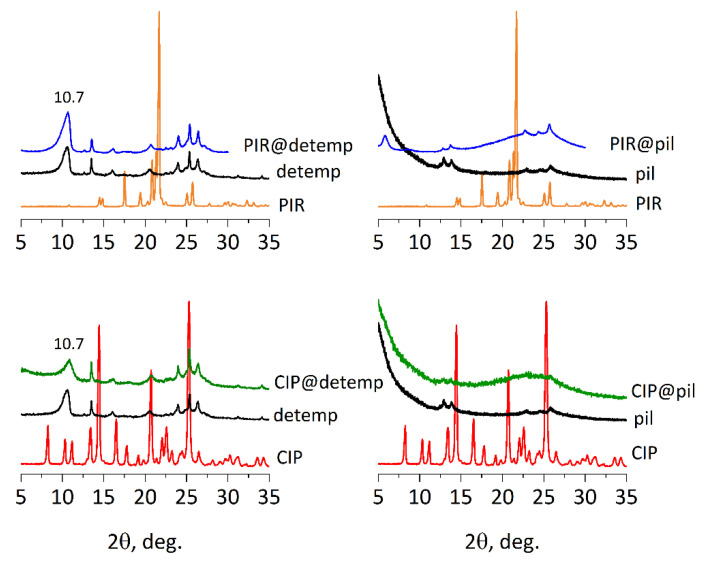
XRD patterns for detemplated (detemp) and pillared (pil) ZSM-55 intercalated with piracetam (PIR) and ciprofloxacin (CIP).

The extent and quality of ZSM-55 modifications were evaluated by XRD and nitrogen adsorption (Figure 3 and Table 1). ZSM-55 was detemplated by reaction with 1 M HCl in methanol at 50 °C for overnight. The obtained sample had very low specific surface area (71 m^2^/g measured after calcination) and sorption capacity (0.05 cm^3^/g), as expected for multilamellar stacking of non-porous (ferrierite) layers. The low angle line position in XRD varied depending on drying conditions indicating variable interlayer space with the degree of hydration. The 002 peak position was variable between 8.3 to 10.5° 2θ (Cu Kα radiation throughout), d-spacing 1.06 to 0.84 nm, and could be shifted to d-spacing values lower than in the complete framework FER/CDO structures (d = 0.91 nm). The pillared material showed interlayer distances expanded to ca. 3.8 nm, with high adsorption capacity of 0.69 cm^3^/g and specific surface area of 1194 m^2^/g. In the XRD patterns for the pillared and detemplated materials, the maxima corresponding to intralayer reflections (hk0) are clearly visible, confirming the preservation of the layers internal structure. These reflections are identified as: (020) at 12.6°, (011) at 13.5°, (031) at 22.5°, (040) at 25.4° 2θ. For the pillared form, they have reduced intensity and are broadened, which may be interpreted as due to the presence of additional silica (pillars) and reduction of the interlayer stacking order in the mesoscale [14].

The observed efficiency of drug intercalation by the applied straightforward solution-solid interaction depended more on the type of drug than the layered zeolite structure—whether detemplated or pillared. On the other hand, the latter influenced the apparent mechanism of release. Piracetam, with a small size and high solubility, was easily incorporated in substantial quantity in both detemplated and pillared ZSM-55 (loading: 21 and 26%, respectively). The sorption of bulky, poorly soluble ciprofloxacin was significantly smaller, 4 and 8% for the detemplated and pillared ZSM-55, respectively. Ciprofloxacin was introduced in acidic conditions, which should make it cationic and favorable to interact with negatively charged zeolite layers [35] but nevertheless did not improve its loading. It is however important that for both types of composites interactions between the drug and the carrier were significant as determined by FT-IR and they influenced the molecular structure of the drug.

XRDs of the solids isolated after reactions of piracetam and ciprofloxacin with detemplated sample did not show increased d-spacing suggesting little or no intercalation between layers (Figure 3). The presumed drug molecule location is on the surface of the outer layers. Loading of the drugs inside the pillared ZSM-55 also did not change the XRD pattern but since the structure is rigid, drug molecules just fill the available spaces (as indicated by increased amount in the sample). In the XRD patterns for composites no reflections characteristic for piracetam or ciprofloxacin are observed, indicating that no drug crystallites with sizes exceeding the standard XRD detection limit, i.e., 2–2.5 nm [36,37,38,39] are present, even with high loading, i.e., 26% by weight of piracetam.

**Figure 4:**
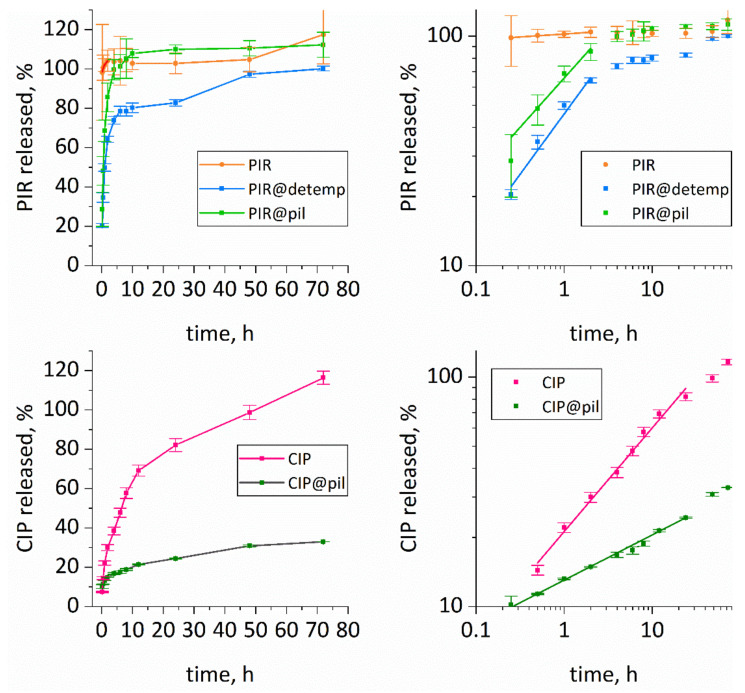
The piracetam (PIR) and ciprofloxacin (CIP) release profiles from detemplated (detemp) and pillared (pil) ZSM-55. The graphs show linear (left panes) and double-logarithmic representation (right panes) with fitting to Korsmeyer-Peppas law. Alternative representations of the data are available in supplementary material (Figure S1).

The release of both drugs to PBS solutions in Franz cells was tested for both detemplated and pillared ZSM-55 samples (Figure 4). The release profiles were different than for the commercial, crystalline forms. In agreement with the literature reports [25,26,34,40], crystalline piracetam dissolved in the PBS solution immediately, while ciprofloxacin was released slowly with full solubilization achieved after 50 h. The piracetam release from both forms of ZSM-55, detemplated and pillared, was slowed down. Half-time of release was achieved after 1.2 and 0.5 h, respectively (Table 2).

For both forms of ZSM-55 the release of piracetam was complete and no drug was irreversibly trapped inside the support. The situation was opposite for ciprofloxacin—only about one-third of the drug was released from the pillared sample; the detemplated sample was not tested due to very low loading of ciprofloxacin. Notably, ciprofloxacin release rate was accelerated, compared to the pure drug.

To understand the dissolution mechanisms from the composites, the release data were fitted using the empirical equation proposed by Korsmeyer and Peppas [41,42]. It contains the following two parameters: *K*, a constant incorporating structural and geometric characteristics of the system, and *n*, the release exponent, indicative of the mechanism of drug release. The release of piracetam may be interpreted in terms of Fickian (*n* = 0.50) and anomalous transport (0.50 < *n* < 1). The latter case is a superposition of two mechanisms—diffusion and degradation, including not only erosion of the matrix but also its relaxation. For piracetam in pillared sample, the *n* value is equal to 0.43, suggesting almost pure diffusion; the pillared zeolite matrix is rigid and does not change (degrade) in the PBS solution, which also holds true for the ciprofloxacin release. Piracetam (and probably also ciprofloxacin), is loaded mainly into the pores, formed by silica pillars between layers. For ciprofloxacin the *n* value is very low (*n* = 0.2), characteristic for sponge-like materials for which the penetration of solvent is easy [42], and may be due to low concentration of the trapped ciprofloxacin leaving more space for the solvent. In the case of detemplated sample with piracetam, the *n* value is much higher, 0.53, suggesting anomalous transport, combining diffusion with matrix flexibility. In the case of this material, piracetam was not intercalated between layers but accommodated in the (meso)pores formed between packs of layers. Such structural arrangement may undergo spatial change when drug molecules are released, which is reflected in the n value, suggesting anomalous transport.

The *K* value is also different for both forms of ZSM-55, equal to 46 and 66, for piracetam release from detemplated and pillared ZSM-55, respectively. Since *K* depends on structural and geometric characteristics of the system, it also reflects the role of different layer arrangement in the mechanism of piracetam release.

**Figure 5:**
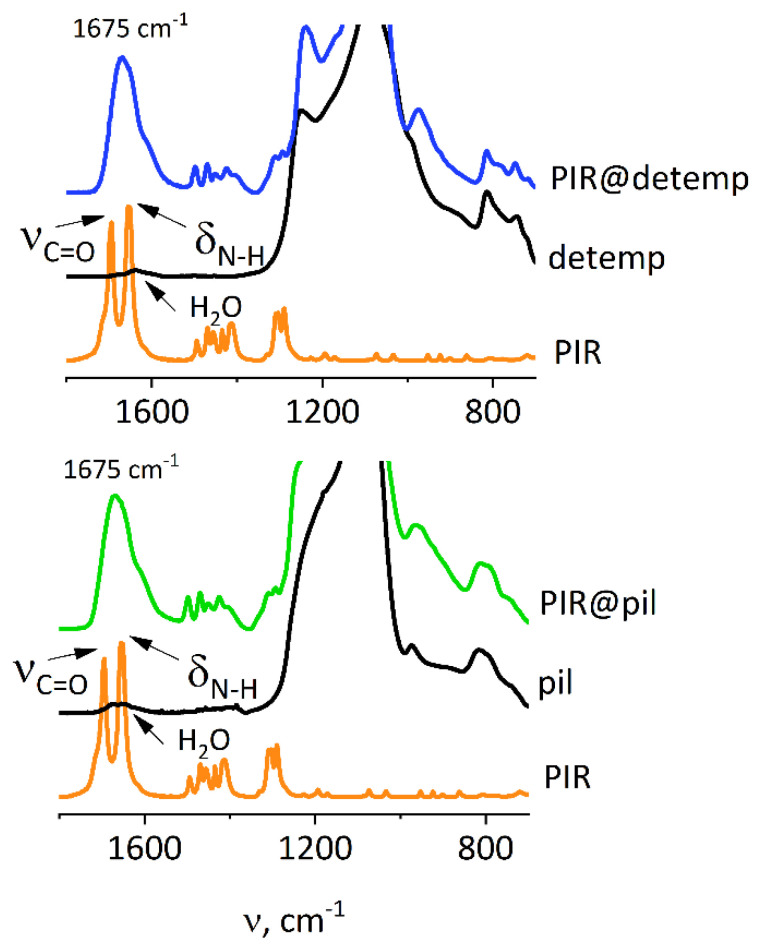
FT-IR spectra for detemplated (detemp) and pillared (pil) ZSM-55 intercalated with piracetam (PIR). Zeolite spectra normalized to 1090 cm^−1^ band (Si-O-Si vibrations), piracetam spectra normalized to the content in respective composite. Alternative representations of the data are available in supplementary material (Figure S2).

**Figure 6:**
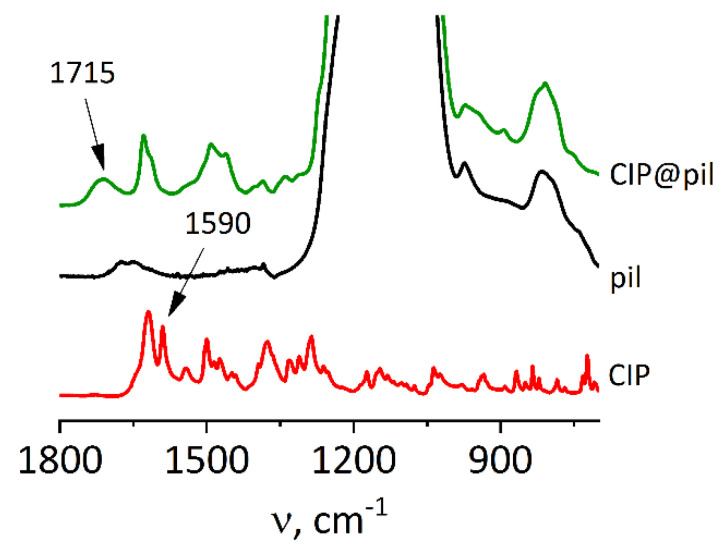
FT-IR spectra for pillared (pil) ZSM-55 intercalated with ciprofloxacin (CIP). Zeolite spectra normalized to 1090 cm^−1^ band (Si-O-Si vibrations), ciprofloxacin normalized to the content in composite. Alternative representations of the data are available in supplementary material (Figure S2).

The rate of drug release should also depend on the interaction between intercalated molecules and the framework of the matrix. FT-IR was used to determine such interactions. Pure zeolites and the corresponding composites were activated in vacuum at 110 °C, i.e., at temperature high enough to remove most of the adsorbed water (small intensity of 1630 cm^−1^ band of H-O-H bending vibrations, Figure 5 and Figure 6) but not the drug. Desorption of water made it possible to observe direct interactions between zeolite and the adsorbed drugs.

Two separate vibrations, which in the crystalline piracetam are attributed to C=O stretching (1695 cm^−1^) and N-H bending (1650 cm^−1^) vibrations, appear in the spectra of the composites as a single, wide band at 1675 cm^−1^ (Figure 5). This is consistent with the formation of hydrogen bonds resulting in blue-shift of the former and red-shift of the latter. It has been proven that upon hydrogen bond formation (both at the C=O and N-H functionalities) the amide I band (predominantly C=O stretching vibrations) is red-shifted, while the amide II band (predominantly N-H bending vibrations) is blue-shifted [43]—two bands may merge together, as in our case. This behavior suggests strong interaction between the carrier and the drug and may be responsible for the modified, slowed down release of piracetam from the ZSM-55 matrix.

The carboxylic acid group located in the quinoline ring of ciprofloxacin can be protonated reversibly. The band at 1715 cm^−1^ (Figure 6) is characteristic of protonated H-bonded form, while the band at 1590 cm^−1^ is due to the deprotonated -COO- group [44]. In the case of crystalline ciprofloxacin the molecules form dimers, the -COOH group is deprotonated and the proton is transferred to the -NH group of the adjacent ciprofloxacin molecule [45]. Thus, in the spectrum of crystalline drug the band at 1590 cm^−1^ is present, while the one at 1715 cm^−1^ is absent. After incorporation of the drug into pillared ZSM-55, the carboxyl group is protonated, probably due to interaction with weakly acidic Si-OH groups present at the layer surface. Ciprofloxacin is therefore hydrogen-bonded to the zeolite matrix, and the strength of this interaction is lower than in crystalline form, where the proton transfer takes place. Better solubility and faster release of ciprofloxacin may be therefore caused by (i) amorphization of the drug, (ii) formation of its protonated form, and (iii) weaker interaction of the ciprofloxacin molecule with the zeolite than with other drug molecules in the crystalline form.

**Figure 7:**
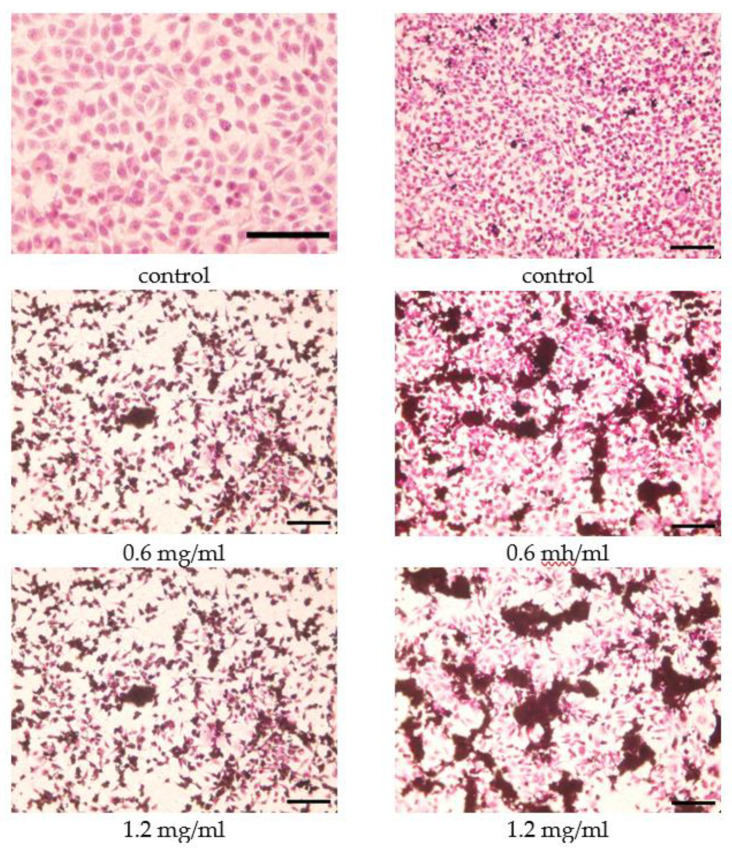
Microscopic images of L929 mouse fibroblasts after 24 h (left column) and 72 h (right column) of incubation with different concentration of ZSM-55. The cells were stained with eosin and hematoxylin. Scale bar: 100 nm.

A viable drug carrier, besides being capable to encapsulate drug molecules and unload the cargo in the patient body, should be non-toxic and biodegradable or not metabolized [46,47]. It is thus imperative to test toxicity of potential carriers and the influence on cells morphology, proliferation and viability. To determine the influence of ZSM-55 on the morphology of the model cells—mouse fibroblasts, microscopic images were taken using an inverted microscope (Figure 7). For these tests the calcined, parent ZSM-55 zeolite was chosen. Previously fixed cells were stained with hematoxylin and eosin. With incubation time, increasing from 24 to 72 h, the number of cells increased, retaining the elongated shape characteristic of mouse fibroblasts, which indicates the lack of toxic effect of the tested carrier.

**Figure 8:**
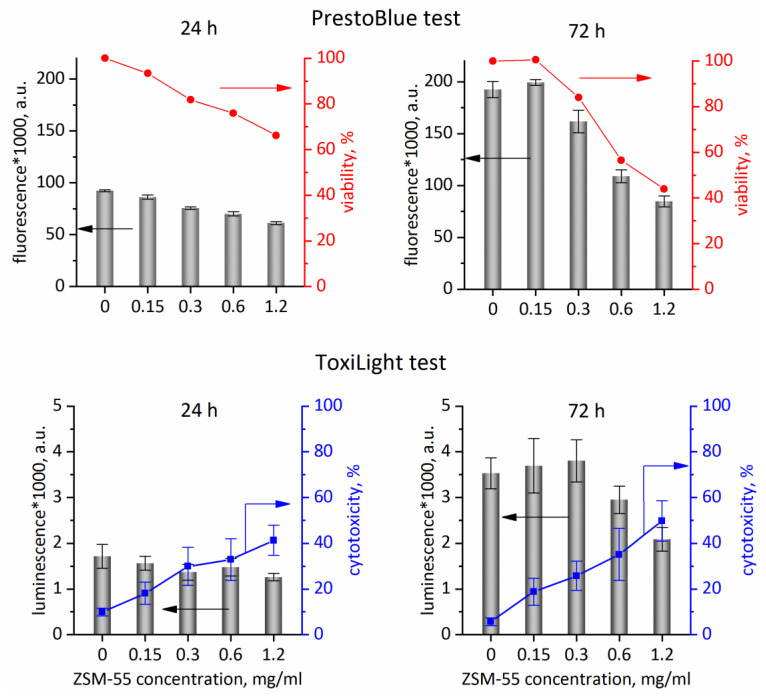
Upper panes: Viability of L929 cells based on the PrestoBlue test: dependence of fluorescence intensity (in relative fluorescence units) on material concentration after 24 h and 72 h of incubation of ZSM-55 material. Lower panes: Proliferation based on ToxiLight test results: luminescence related to concentration of living cells (bars) and cytotoxicity (lines) dependence on the concentration of ZSM-55 after 24 h and 72 h of incubation.

Microscopic observations are only qualitative, while quantitative data are supplied by ToxiLight™ test (toxicity effect) and PrestoBlue test (providing proliferation rate and viability of cells). After 24 h of incubation the cells relative viability depends almost linearly on the ZSM-55 concentration (Figure 8, upper panes). For the highest ZSM-55 concentration (1.20 mg/mL) the cells viability was reduced to ca. 65%. After extension of the incubation time to 72 h, the viability decreased to ca. 45% for the highest zeolite concentration, compared to untreated cells. For the control, fluorescence intensity (measure of the concentration of living cells) measured after 24 and 72 h of incubation increased by a factor of two, while for the highest ZSM-55 concentration fluorescence intensity increased by a factor 1.4. That means that even at the highest concentration, ZSM-55 only inhibited cells viability but did not cause complete inhibition of proliferation.

Analysis of the data obtained using the ToxiLight test (Figure 8, lower panes) confirmed the results obtained with the PrestoBlue test. After 24 h, for the highest applied concentrations of tested material (1.2 mg/mL), a reduced number of cells and an increased level of cytotoxicity was observed (increase by ca. 30% between control and the highest ZSM-55 concentration). After 72 h of incubation, the toxicity of the ZSM-55 sample increased only slightly compared to the level reached after 24 h (41 vs 49% for the highest ZSM-55 concentration), however the number of cells after 72 h was higher than after 24 h, which means that even high material concentration, equal to 1.2 mg/mL did not stop proliferation, only slowed it down. For the control, luminescence (measure of the concentration of living cells) measured after 24 and 72 h of incubation increased by a factor of two, while for the highest ZSM-55 concentration it increased by a factor of 1.7.

The results, obtained in two independent tests show that ZSM-55, in moderate concentrations may be safely used as a carrier of drug molecules, not having negative effect on the cells viability or proliferation rate.

## 3. Materials and Methods

### 3.1. Sample Preparation

ZSM-55 was prepared and transformed into detemplated, swollen and pillared forms as described earlier [14]. In a typical preparation, the mixture of 1.36 g boric acid in 120 g of water, 10 g of 50% NaOH, 40 g choline chloride and 65 g of colloidal silica Ludox LS30 was heated in a Teflon-lined autoclave at 150 °C for 90 h with rotation. For template extraction (detemplation) 5 g of ZSM-55 was contacted with 100–150 mL of the 1:10 mixture of concentrated HCl and methanol at 50 °C, overnight. To obtain the pillared product, a detemplated sample was swollen at room temperature with a 50/50 mixture of solutions of 25% hexadecyltrimethylammonium chloride and hydroxide (solid to solution ratio 1:20 *w*/*w*). After swelling, the solid was centrifuged, washed with water and air-dried at 65 °C. Pillaring was carried out by mixing the swollen material with TEOS (1:100 *w*/*w*), stirring overnight at room temperature and solid isolation by centrifugation and air-drying. Calcinations were carried out for 6 h at 550 °C with heating ramp of 2 °C/min. All chemicals were obtained from Sigma Aldrich Poland (Wrocław, Poland).

### 3.2. Drugs Intercalation

Ciprofloxacin intercalation: 0.5 g of the ZSM-55 derivative, detemplated or pillared, was stirred with 50 cm^3^ of 0.1 M HCl containing 0.5 g of ciprofloxacin on a magnetic stirrer for 24 h at room temperature, washed with small amount of 0.1 M HCl and air-dried at room temperature. Piracetam intercalation: 0.5 g of the ZSM-55 derivative, detemplated or pillared, was mixed with 10 cm^3^ of 10% aqueous solution of the drug on a magnetic stirrer for 24 h at room temperature, washed with small amount of water and air-dried at room temperature.

Loading capacity (LC%) and loading efficiency (LE%) were calculated on the basis of the following formulas:

(1) $$\mathrm{LC}=\frac{\mathrm{total}\;\mathrm{entrapped}\;\mathrm{drug}\;\mathrm{mass}}{\mathrm{zeolite}\;\mathrm{mass}}\cdot 100\%$$

(2) $$\mathrm{LE}=\frac{\mathrm{total}\;\mathrm{drug}\;\mathrm{added}-\mathrm{free}\;\mathrm{drug}\;\mathrm{in}\;\mathrm{supernatant}}{\mathrm{total}\;\mathrm{drug}\;\mathrm{added}}\cdot 100\%$$

### 3.3. Drugs Release

The drug release study was carried out in Franz cells [15]. The donor part of the cells (containing the composite) was separated from the acceptor part (solution) by a 0.8 μm cellulose acetate filter (Sartorius, Göttingen, Germany), simulating a contact layer. Approximately 5 mg of the test material were placed on the filter, the acceptor part was filled with 5 mL of phosphate buffered saline (PBS) at pH 7.4 [16]. The experiment was carried out at constant temperature of 37 °C (simulating human body temperature) with continuous mixing at 200 rpm. Samples were taken from a cell using a 0.2 mL syringe at various time intervals (0.25; 0.5; 1; 1.5; 2; 4; 6; 8; 10; 24; 48; 72 h). To maintain constant volume, the system was supplemented each time with the same amount (0.2 mL) of fresh PBS solution. Three tests were carried out for each composite in order to obtain reliable and reproducible results. The control was performed by releasing pristine crystalline drug in the amount corresponding to the drug content in the tested materials.

The total content of piracetam and ciprofloxacin in the tested materials was determined by mixing the composites (5 mg) in a PBS solution (20 mL) for 72 h at 37 °C. The resulting solution was then centrifuged to separate the solid from the solution. A 2 mL sample was taken, diluted and concentration of the drug released into the solution was measured using UV/Vis spectroscopy (Lambda spectrometer, Perkin-Elmer, Waltham, MA, USA). The percentage of the drug obtained that way was considered as the baseline (100%) of the content of the drug in a given carrier.

Release curves were fitted to experimental points by Origin software (Origin 2018, Northampton, MA, USA) using the Korsmeyer-Peppas law [48] to determine the mechanism of molecule release—estimating the exponent *n* in Equation (3):

(3) $$\frac{M_{i}}{M_{\infty }}=Kt^{n}$$

where *M*_∞_ is the amount of drug at the equilibrium state, *M_i_* is the amount of drug released over time *t*, *K* is related to the release velocity constant, and *n* is the exponent of release (related to the drug release mechanism) in function of time *t*. The exponent was determined from the portion of release curve until the deflection point in order to provide sufficient number of measured points although it exceeded the cutoff at 60% of the released drug (by weight), recommended for the application of this law. The enhancement in the number of points did not affect the value of the exponent n [49], which is best visualized by the linear course of the release curves in double logarithmic scale (Figure 4).

### 3.4. Physicochemical Characterization

The structure and crystallinity of obtained samples were determined by X-ray powder diffraction (XRD) using a MiniFlex diffractometer (Rigaku, The Woodlands, TX, USA) in reflection mode, using CuK_α_ radiation (λ = 0.154 nm). The XRD patterns were collected with steps of 0.02°.

Nitrogen adsorption-desorption isotherms were determined by the standard method at −196 °C (liquid nitrogen temperature) using an ASAP 2020 (Micromeritics, Norcross, GA, USA) static volumetric apparatus. Before adsorption the samples (ca. 200 mg) were outgassed at 110 °C overnight using turbomolecular pump to remove adsorbed water.

For FT-IR studies, the samples in the form of thin layer deposited on silicon wafers (ca. 10 mg) were dehydrated at 110 °C under vacuum in a custom-made IR cell enabling in situ treatment at variable temperatures. IR spectra were recorded on a Tensor 27 spectrometer (Bruker, Ettlingen, Germany) equipped with a MCT detector, working with spectral resolution of 2 cm^−1^.

### 3.5. Cytotoxicity and Cells Viability

Proliferation of cells and the cytotoxicity effect of the studied material was determined by ToxiLight™ assay (Lonza, Greenwood, SC, USA). The test was used to calculate the concentration of adenylate kinase (AK) in the supernatant (representing damaged cells) and lysate (representing intact adherent cells). Viability of cells was examined by resazurin-based reagent PrestoBlue™ (Invitrogen, Carlsbad, CA, USA). The fluorescent product of the resazurin reaction and luminescence from luciferase in the presence of AK (ToxiLight™ test) were detected using a POLARstar Omega microplate reader (BMG Labtech, Ortenberg, Germany). Cells morphology was observed under inverted microscope CKX53 (Olympus, Tokyo, Japan) after eosin/hematoxylin staining.

ToxiLight™ tests were performed after 24 and 72 h of incubation of fibroblasts with the tested materials, and the assay procedure was performed according to the producer protocol.

## Figures and Tables

**Table 1:** Basic textural parameters for detemplated and pillared ZSM-55. External surface area (S_ext_) and pore volume (V_T-plot_) were calculated by the t-plot method, combining micropores and small mesopores, while total volume (V_tot_) was calculated at p/p^0^ = 0.95.

ZSM-55	S_BET_, m^2^/g	S_ext_, m^2^/g	V_T-plot_, cm^3^/g	V_tot_, cm^3^/g
detemplated	71	17	0.02	0.05
pillared	1194	72	0.56	0.69

**Table 2:** Exponent of release *n*, kinetic constant *K* and half-time of release *t*_1/2_ for fitted Korsmeyer-Peppas release curves (Equation (3)) and coefficient of determination R^2^ for the double-logarithmic fitting presented in Figure 4. Drug content determined by elemental analysis and UV/Vis experiments of total release of the drug from the composites (see Section 3 for details). Loading capacity (LC) and loading efficiency (LE%) were calculated using formulas presented in Section 3 (Equations (1) and (2)).

ZSM-55	Drug	*K*	*t*_1/2_, h	*n*	R^2^	Drug Content, %		LC, %	LE, %
						Analysis	UV/Vis		
	CIP	21	921	0.45 ± 0.02	0.990				
detemplated	CIP					4		4.2	4.2
pillared	CIP	13	6.7	0.20 ± 0.002	0.999	8	9	9.9	9.9
	PIR	102	0	0.02 ± 0.003	0.975				
detemplated	PIR	46	1.2	0.53 ± 0.06	0.977	21	18	24.2	12.1
pillared	PIR	66	0.5	0.43 ± 0.07	0.949	26	26	35.1	17.6

## Supplementary Materials

The following are available online. Figure S1: The piracetam (PIR) and ciprofloxacin (CIP) release profiles from detemplated (detemp) and pillared (pil) ZSM-55; Figure S2: FT-IR spectra for detemplated (detemp) and pillared (pil) ZSM-55 intercalated with piracetam (PIR) and ciprofloxacin (CIP).
