# Supplementary material for: Detemplated and Pillared 2-Dimensional Zeolite ZSM-55 with Ferrierite Layer Topology as a Carrier for Drugs
Source: Molecules. 2020 Jul 31;25(15):3501. doi: 10.3390/molecules25153501 (PMC7435734; doi:10.3390/molecules25153501)
Supplement: Supplementary file 1 [file molecules-25-03501-s001.pdf]

Supplementary Material

Detemplated and pillared 2-dimensional zeolite ZSM-55 with ferrierite layer topology as a carrier for drugs

Weronika Strzempek, Aleksandra Korzeniowska, Andrzej Kowalczyk, Wieslaw J. Roth and Barbara Gil *

Faculty of Chemistry, Jagiellonian University, Gronostajowa 2, 30-387 Kraków, Poland
* Correspondence: gil@chemia.uj.edu.pl;

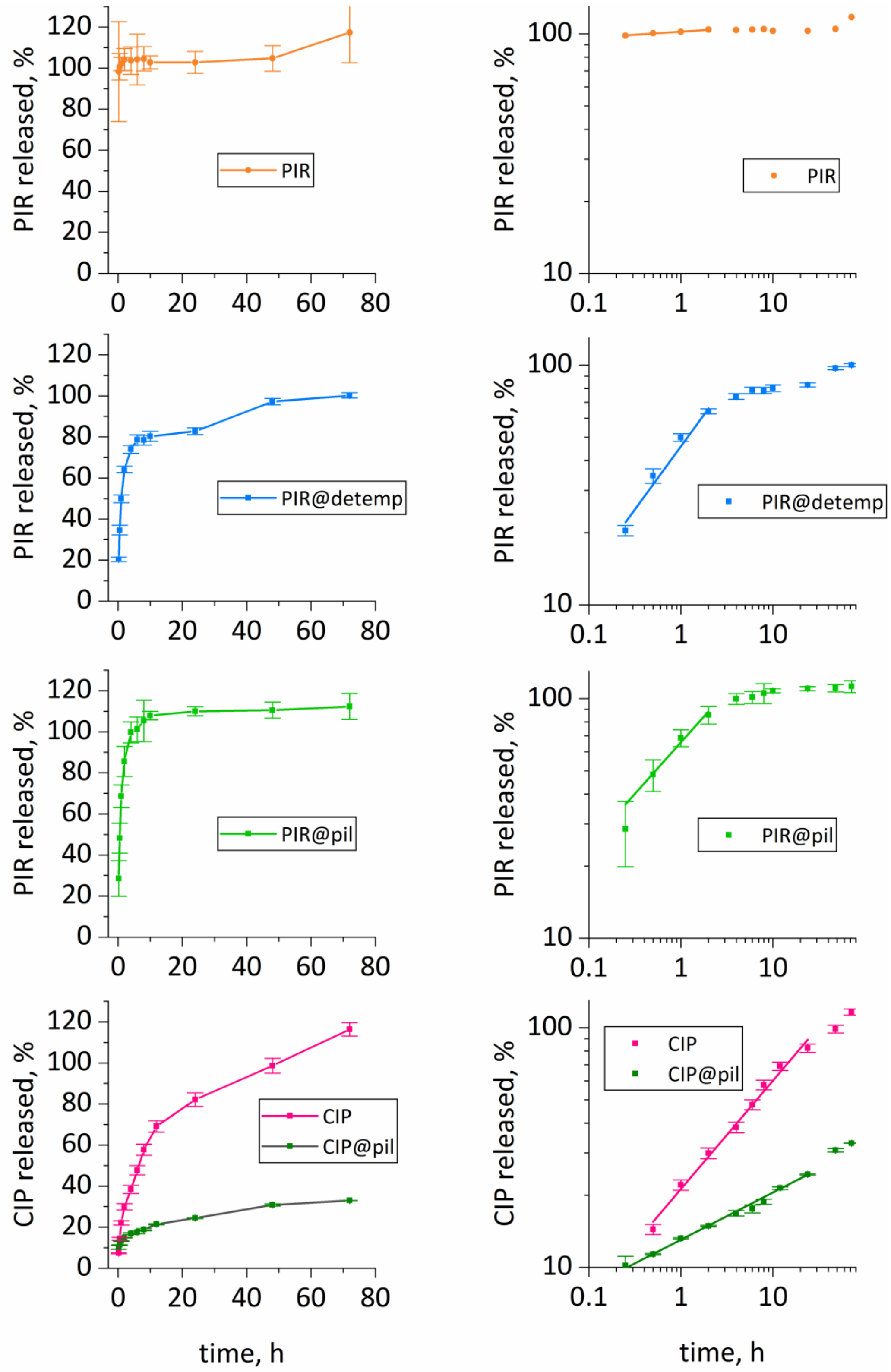

Figure S1 The piracetam (PIR) and ciprofloxacin (CIP) release profiles from detemplated (detemp) and pillared (pil) ZSM-55. The graphs show linear (left panes) and double-logarithmic representation (right panes) with fitting to Korsmeyer-Peppas law. Alternative representation of Figure 4.

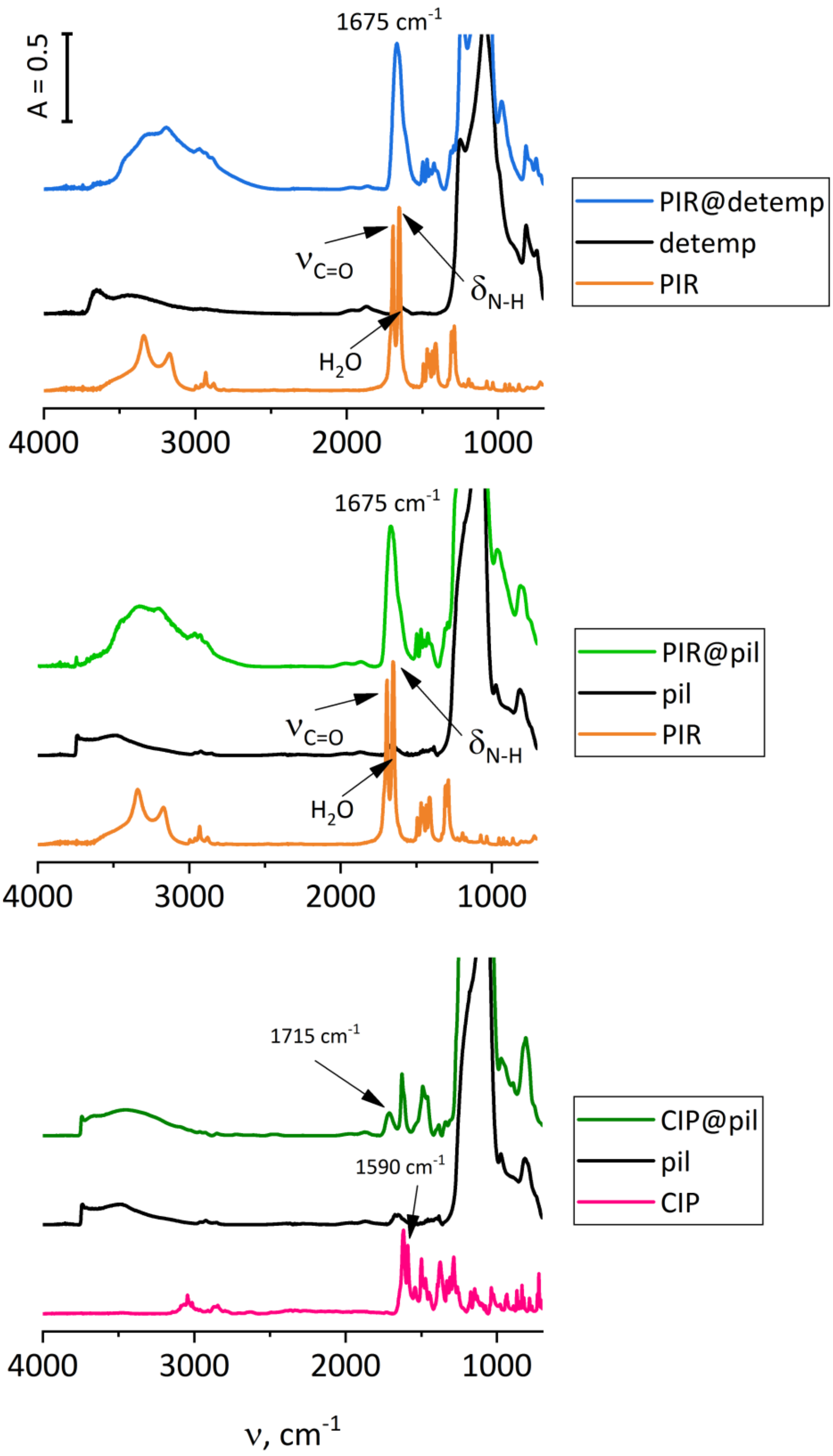

Figure S2. FT-IR spectra for detemplated (detemp) and pillared (pil) ZSM-55 intercalated with piracetam (PIR) and ciprofloxacin (CIP). Zeolite spectra normalized to 1090 cm^{-1} band (Si-O-Si vibrations), piracetam and ciprofloxacin spectra normalized to the content in respective composite. Alternative representation of Figures 5 and 6.
